# Measuring disparities in police use of force and injury among persons with serious mental illness

**DOI:** 10.1186/s12888-021-03510-w

**Published:** 2021-10-12

**Authors:** Ayobami Laniyonu, Phillip Atiba Goff

**Affiliations:** 1 University of Toronto, Toronto, Ontario Canada; 2 Yale University, New Haven, Connecticut USA; 3 Center for Policing Equity, New Haven, CT USA

## Abstract

**Objectives:**

To measure disparities in experience of police use of force and injury among persons with serious mental illnesses.

**Methods:**

We gathered novel police use of force and suspect injury data from 2011 to 2017 from a nonrandom sample of nine police departments in the United States and used synthetic methods to estimate the share of the local population with serious mental illness. We estimate disparities using multi-level models estimated in a Bayesian framework.

**Results:**

Persons with serious mental illness constitute 17.0% of use of force cases (*SD* = 5.8) and 20.2% of suspects injured in police interaction (*SD* = 9.0) in sample cities. The risk that persons with serious mental illness will experience police use of force is 11.6 times higher (95% CI, 10.7–12.6) than persons without serious mental illness. Persons with serious mental illness are also at a higher risk of experiencing injury, 10.7 times (95% CI, 9.6–11.8), relative to persons without serious mental illness. These relative risk ratios are several times larger than racial and ethnic disparities estimated in the same cities.

**Conclusion:**

Persons with serious mental are at a significantly elevated risk of experiencing police use of force and injury in police encounters than the general public. The disparities we estimate are several times higher than racial/ethnic disparities in force and injury. Efforts to reform police practices and reimagine public safety in the United States should address significant disparities in police use of force against those with serious mental illness.

**Supplementary Information:**

The online version contains supplementary material available at 10.1186/s12888-021-03510-w.

## Background

The current discussion of disparities in police contact and use of force largely revolves around racial disparities. Empirical work suggests that Black people are disproportionately more likely than White people to come into contact with the police, experience force, and to suffer adverse psychological outcomes as a result of encounters [1–3].

But racial inequality is not the only dimension on which disparate contact exist and contacts need not necessarily result in force or injury. For instance, while Black populations experience elevated levels of police contact and force, contact may be elevated but force *less* likely among elderly persons who are more likely to call 911 but are not perceived to be threatening to officers. Similarly, contact may be elevated, but force reduced among persons with serious mental illness (PwSMI) who are more likely to engage in behaviors that produce calls to 911 but may be viewed by police as less deserving of blame for their behaviors [4].

It is not clear whether or to what extent PwSMI experience disparate police use of force. Past research suggests that police generally hold that PwSMI are less responsible for crimes they might commit and more deserving of care than persons without serious mental illness [4] and it may be that officers’ responses to PwSMI are more protective than their responses towards the broader public. In one study, law enforcement officers reported distress or unhappiness when a lack of available beds forced them to jail PwSMI, rather than facilitate their intake into hospitals or psychiatric care facilities [5]. Similarly, past research suggests that law enforcement officers occasionally engage in a practice called ‘mercy booking’, wherein officers jail PwSMI for low level misdemeanors in situations where alternative treatment in healthcare facilities is unavailable [6]. Although this particular practice might contribute to the overrepresentation of PwSMI in jails [6], if the motivation of law enforcement officers is to protect PwSMI from harm, then we should not expect officers to treat PwSMI in a manner prone to result in injury.

We may also reasonably expect, however, for PwSMI to experience especially harsh forms of policing. Deinstitutionalization and significant cuts to federal spending on public health increased contact between PwSMI and law enforcement and PwSMI may be more likely to experience force and injury as a result [7]. PwSMI may also be more likely to resist police officers in encounters, leading to higher rates of force and injury, or force and injury may be a consequence of officer ignorance of how serious mental illness can affect behavior [8, 9]. PwSMI may also be more likely to reside in areas with high levels of violent crime or to be unhoused and therefore more likely to be exposed to officers and force than persons without SMIs [10].

Historically, efforts to estimate disparities in police use of force against PwSMI have been hampered by a lack of police use of force data that include information on officer perceptions of mental impairment and data on the number of PwSMI in a local area. To address the first issue, this study utilized unique data from nine large police departments in which police officers note their perception of mental impairment of persons who experience force or injury. To address the second issue, it used synthetic methods to generate estimates of the local share of the populations with serious mental illnesses (SMIs).

Although police officers may view PwSMI as more vulnerable than other residents and may therefore refrain from using force relative to persons without SMIs, we hypothesized that the combined effects of officer stereotypes, the behavior of PwSMI in encounters, and greater exposure of PwSMI to officers will lead to large disparities in the rates PwSMI experience force. If the rates at which police use force against PwSMI are different than the general population, how should we interpret those findings? To provide context for our results, we compared disparities between persons with and without SMI to disparities between Black, Latinx, and White residents in the same jurisdictions. Non-White/White disparities in policing outcomes are by far the most commonly studied and can serve as a kind of benchmark for the magnitude of any disparities observed between persons with and without SMI.

## Methods

### Data

Prior to commencement of the study, Ethics Approval was granted from the Institutional Review Board at John Jay College of Criminal Justice. All methods were performed in accordance with our protocol as well as all other relevant guidelines and regulations. Informed consent was gained from participants prior to the commencement of the study.

We compiled data on police use of force, suspect injury, and suspect mental health status from a total of nine police departments. Data from six departments came from the National Justice Database, a large repository of police data housed at the Center for Policing Equity. Participation in this repository is confidential and none of these six departments are named in this manuscript. All departments included in the analysis are located in moderately sized cities, which ranged in population from approximately 300,000 to approximately 1,000,000 people in 2017. In addition to data from these six anonymous police departments, the analysis included publicly available data on police use of force, suspect injury data, and suspect mental health status from New Orleans, Louisiana, Dallas, Texas, and Los Angeles, California. In total, our dataset constituted 28,649 police use of force events occurring between 2011 and 2017.

Police department in all sample cities had implemented trainings designed to improve police interaction with mentally ill suspects. Literature suggests that when officers have been trained to identify PwSMI they do so with a moderate to high degree of accuracy, and that this crisis intervention training (CIT) significantly improves their ability to identify symptoms associated with SMI [11, 12]. Nevertheless, our reliance on officer classification introduces error into our estimation of disparities. We note, however, that police are more likely to fail to identify PwSMI rather than attribute SMI to individuals without it [11]. This suggests that a disparity which identifies that individuals with SMI are more likely to experience use of force will be conservative, as official police data underestimates use of force against individuals with SMI and overestimates use of force against those without it.

We gathered survey data on SMI from the National Comorbidity Survey Replication (NCS-R), a nationally representative multi-stage survey on the prevalence and correlates of mental disorders in the United States [13, 14]. Interviews were conducted face-to-face between 2001 and 2003 among English-speaking heads of household aged 18 and over. While the NCS-R surveyed 9282 respondents, this analysis utilized a subset of those respondents (*N* = 5493) who completed the long version of the survey. The longer version of the survey included additional questions measuring demographic characteristics that were used to develop the multivariate logistic regression of SMI.

Aggregate level city and Census tract-level data were pulled from the American Community Survey (ACS). Throughout the analyses, yearly ACS data were pulled to correspond to the year for which disparities in police use of force and suspect injury were being analyzed. For example, disparities estimated from 2016 police data were generated using 2016 ACS data.

### Operationalization of serious mental illness

Our operationalization of SMI from the NCS-R followed methodologies from previous research that coded individuals as suffering from SMI if they met criteria for diagnosis, functional impairment, and duration [15, 16]. Accordingly, individuals were coded as suffering from SMI if they met each of the following three criteria. First, individuals must have met the Composite International Diagnostic Interview (CIDI) diagnosis for any of the following mental illnesses: bipolar I, bipolar II, mania, major depressive disorder, agoraphobia, generalized anxiety disorder, post-traumatic stress disorder, hypomania, specific phobia, social phobia, or seriously attempted suicide within the past 12 months. Second, individuals diagnosed with any of these conditions must have: a) reported being unable to function due to that condition for at least 120 out of the past 365 days or b) rated the magnitude of their impairment at work, at home, in relationships and in their social lives due to the condition as at least 7 out of 10. Third individuals must have reported having the disorder for at least 12 months.

### Variable coding

The small area estimation strategy used here required that the variables used to generate predictors of SMI in the individual level data set are contained in the aggregate ACS data and coded in the same fashion. Based on extant work, we predicted SMI as a function of age, gender, race, marital status, employment status, educational attainment, and poverty index [15]. Codings are presented in the online Additional file 1. Identical data were pulled from the ACS and were coded in the same fashion as the individual data.

### Small area estimation

Statistical estimation of disparities in police use of force relies on a baseline against which to measure disproportionality. Observed disparities in police use of force may reflect the greater exposure that these populations have to the police or other ecological factors (such as crime rates), and statistical estimation of disparity must control for this possibility. No federal agency, however, conducts a census of the share of the population with SMI at the state, county, or tract level. And, while nationally representative surveys provide reliable estimates of the prevalence of SMI across the United States, the sampling frame of these surveys rarely contain enough respondents from any particular city or county to estimate the number of PwSMI at that level.

We used *synthetic estimation* to approximate the share of the population with and without SMI at the neighborhood, police precinct, and city levels. Synthetic estimation is a procedure for producing estimates at relatively small geographies that applies data from nationally representative surveys to large administrative data [17]. We employed a model-based approach, which first estimated prevalence of SMI among subpopulations defined by race, age, marital status, and other socioeconomic and demographic characteristics using data from a nationally representative survey and then projected these estimates onto aggregate data from the US Census Bureau [15, 16, 18–20].

Small area estimation of the share of the population with and without SMI proceeded as follows. First, a multivariate logistic regression using the individual level predictors of SMI was generated from the NCS-R. Regression coefficients from this model were then applied to police-precinct and Census tract level data to generate logit scores. Logit values for these areal units were then converted to probabilities, which were then multiplied by the ACS estimate of the total population over the age of 18 to produce estimates of the population with SMI.

### Estimation of disparities

Disparities in use of force and injury between individuals with and without SMIs were estimated using hierarchical negative binomial models, estimated in a Bayesian framework. Specifically, we model:

$$ {y}_{vti}\sim Negative\ Binomial\left({n}_{vti}{e}^{u+{\alpha}_v+{\delta}_t+{\beta}_i},\phi \right) $$

$$ {\beta}_i\sim Normal\left(0,{\sigma}_{\beta}^2\right) $$

where *y*_*vti*_ are use of force events/injuries for populations defined by different levels of vulnerability *v* in different years *t* across different tracts/police precincts *i*. We model the distribution of *y*_*vti*_ as following a negative binomial distribution and set *n*_*vti*_—the share of the population with and without SMI at each geography in each year—as the corresponding offset or measure of exposure. The parameter *ϕ* controls the shape of the negative binomial distribution and is estimated from the data. Our key coefficient *α*_*v*_ measures the effect that status as a PwSMI has on the likelihood of use of force/injury, while *δ*_*t*_ measures year specific effects and *β*_*i*_ captures location specific effects. We assume these location specific effects follow a normal distribution with mean 0 and standard deviation ${\sigma}_{\beta}^2$ (which is estimated from the model). We set weakly informative priors for all model coefficients and estimate the posterior distribution of model parameters in the R programming language using the brms package [21]. Convergence statistics are displayed in the online Additional file 1.

In addition to estimating the disparity in police use of force and injury for PwSMI, we also estimate disparities in police use of force against Black, Latinx, and White residents, so as to better contextualize the size of the disparity. We estimate these disparities using a similar model as above, but where *y*_*rti*_ are use of force events/injuries for populations defined by race *r* and where *n*_*rti*_ is the share of the population belonging to each racial group, and *α*_*r*_ measures the effect of race on likelihood of use of force/injury.

## Results

**Fig. 1:**
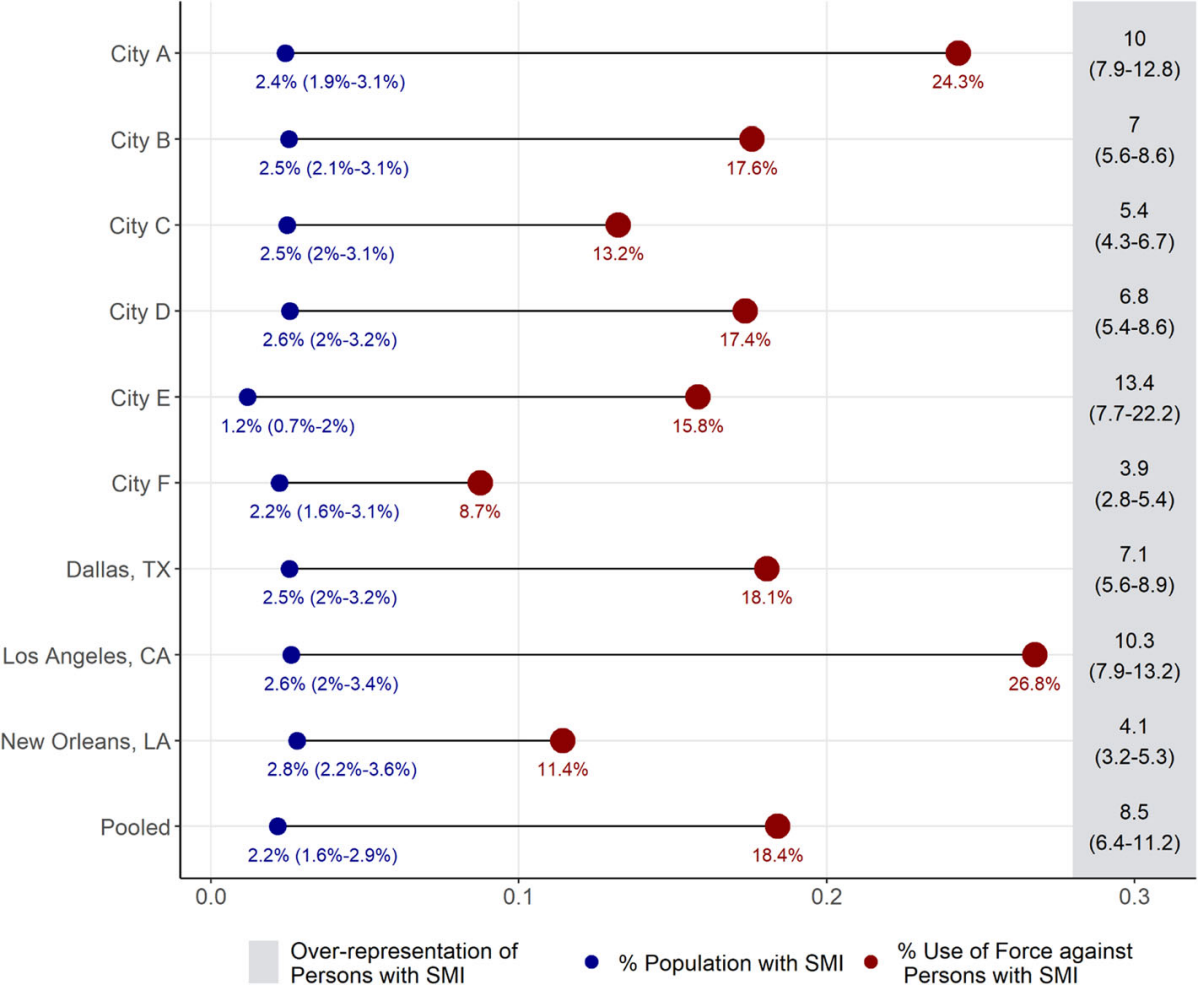
City-level disparities in use of force among persons with serious mental illness (SMI)

Figure 1 depicts raw, city-level disparities in police use of force, with results from the pooled sample at the bottom. For each city, the estimated prevalence of SMI in the population is plotted in blue and on the left, while the share of use of force incidents committed against PwSMI is plotted in red on the right. On the far-right hand side, Fig. 1 depicts the raw measure of how overrepresented PwSMI are in use of force incidents, calculated as the number of force incidents against PwSMI for the corresponding city over the estimated size of the population with SMI. For simplicity, Fig. 1 summarizes data from the most recent year available for each city. The disparities calculated from the most recent year in each city is, however, representative of disparities across time.

Overall, the results plotted in Fig. 1 suggest that PwSMI are significantly overrepresented in use of force incidents. Specifically, while the prevalence of SMI in the sample cities range from 1.3 to 3.1%, the share of use of force incidents directed towards individuals with SMI ranges from 8.7% to 26.8%. Relative to the prevalence of SMI in the population of these cities, PwSMI are between 3.5 to 12 times overrepresented in use of force data.

**Fig. 2:**
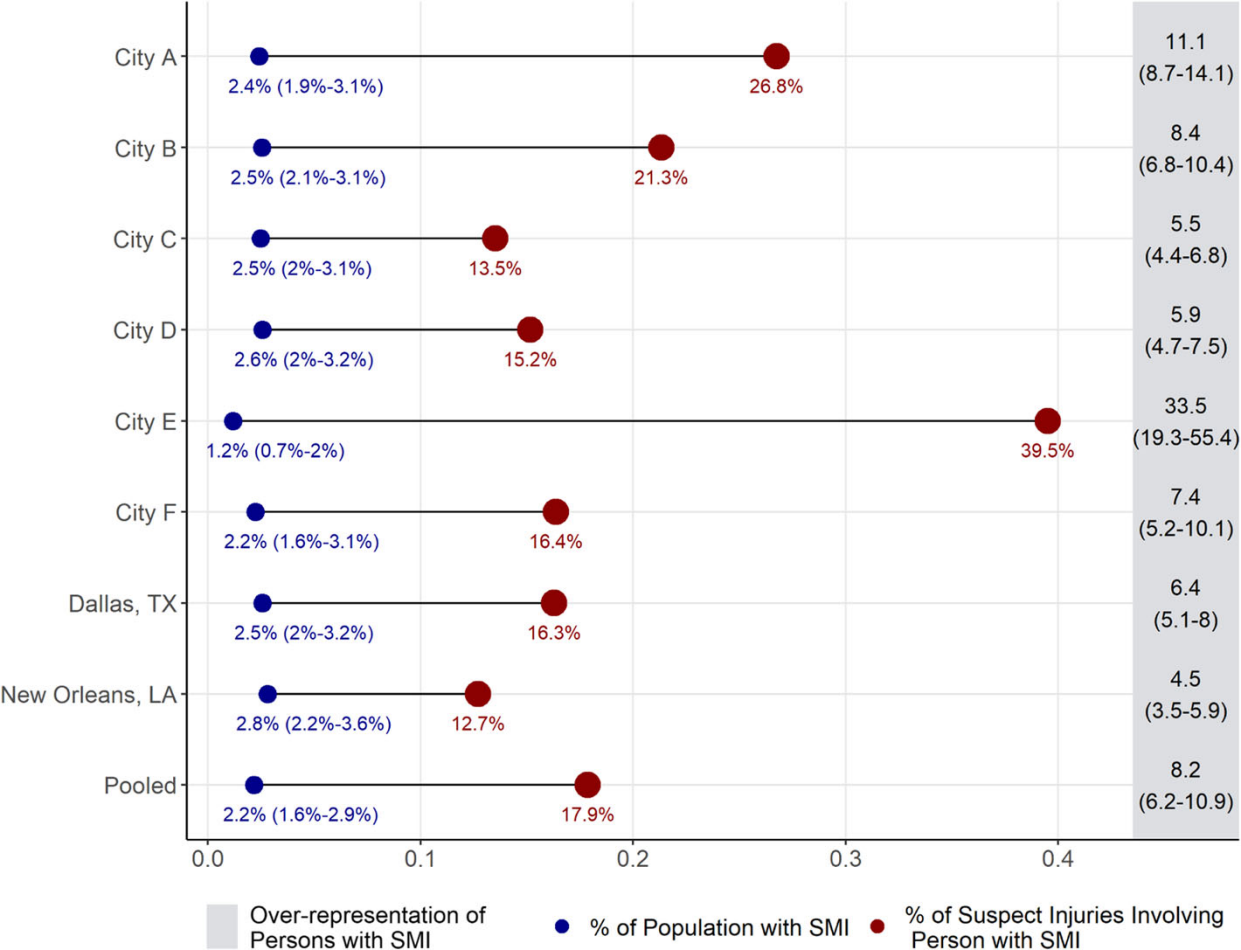
City-level disparities in injuries among persons with serious mental illness (SMI)

Figure 2 depicts the raw, city-level disparity in suspect injury in a similar fashion. Note that data collected from Los Angeles did not include information on injuries experienced by PwSMI and is therefore omitted from Fig. 2. As with the results presented in Fig. 1, the results here suggest significant overrepresentation of individuals in injury related incidents relative to their presence in the population. Estimated overrepresentation of PwSMI generally surpass their general overrepresentation in use of force cases, with results suggesting that individuals with SMI are 4 to 30 times overrepresented in use of force cases relative to their share of the population.

While indicative of a significant overrepresentation of PwSMI in adverse interactions with police officers, these city-wide rates do not tell the entire story as they could be the consequence of the distribution of PwSMI across parts of the city. If, for example, a significant share of the population with mental illness lives in neighborhoods where crime is high or officers are especially proactive, then the results may be driven by where PwSMI are located in cities. The results from the hierarchical negative binomial models presented in the following section take these neighborhood-level factors into account.

Table 1 presents coefficient estimates from our negative binomial model, run both on Census tracts and police-precincts. Data were drawn from 6 cities where incidents were geo-locatable. Results estimated at both levels of aggregation suggest significant disparities in police use of force against individuals with SMI. Tract-level estimates suggest that PwSMI are 11.6 times more likely to experience use of force than persons without SMIs and 10.7 times more likely to experience injury. Estimated disparities at the precinct level mirror those at the tract level. Here, PwSMI appear to be 10.2 times more likely than persons without SMI to experience use of force and 11.6 times more likely to experience injury.

**Table 1:** Estimated disparity in police use of force and suspect injury for persons with serious mental illness (PwSMI) in Census tracts and police precincts relative to persons without serious mental illness (relative risk)

	Use of Force		Suspect Injury	
	Census Tract	Police Precinct	Census Tract	Police Precinct
PwSMI	11.59	10.18	10.70	11.59
	(10.7, 12.55)	(8.85, 11.82)	(9.58, 11.82)	(9.87, 13.74)
2012	0.87	0.90	0.84	0.89
	(0.74, 1.02)	(0.64, 1.27)	(0.67, 1.06)	(0.61, 1.27)
2013	1.34	1.38	1.40	1.42
	(1.13, 1.57)	(0.96, 1.97)	(1.12, 1.77)	(0.97, 2.05)
2014	1.32	1.32	1.34	1.34
	(1.13, 1.57)	(0.91, 1.88)	(1.06, 1.68)	(0.9, 1.95)
2015	1.20	1.27	1.31	1.31
	(1.02, 1.42)	(0.9, 1.8)	(1.05, 1.63)	(0.9, 1.92)
2016	1.02	1.46	0.90	1.34
	(0.84, 1.22)	(0.96, 2.2)	(0.7, 1.15)	(0.84, 2.1)
σ_β_	1.61	1.12	1.7	1.22
Φ	1.23	3.27	0.85	2.92
N	5978	268	5978	268

*Notes:* Data from the National Justice Database and several public police departments. Numbers in parentheses give 95% credible intervals

How can we substantively make sense of a disparity of this size? Using similar methods, extant studies have estimated disparities in police stops and use of force against Black people and other minority groups in the United States. We compare the disparities we estimate among individuals with serious mental illness to disparities in use of force by race and ethnicity, using a modeling strategy similar to that presented in Model 1. Instead of modeling use of force and injury for persons with and without SMIs, however, we model the number of force and injury experienced by Black, Latinx, and White people.

How does our estimate of the disparity in use of force and injury among PwSMI compare to disparities in race? Table 2 depicts the likelihood that Black and Latinx people will experience use of force or injury, relative to the share of both the Census tract and the police-precinct. Results from this analysis suggest that the disparities estimated in Table 1 are significantly higher than those estimated between White and non-White groups in the cities analyzed here.

**Table 2:** Estimated disparity in police use of force and suspect injury for Black and Latinx Americans in Census tracts and police precincts relative to White Americans (relative risk)

	Use of Force		Suspect Injury	
	Census Tract	Police Precinct	Census Tract	Police Precinct
Black	3.13	3.67	2.59	3.25
	(2.86, 3.39)	(3.22, 4.14)	(2.32, 2.92)	(2.83, 3.74)
Latinx	0.96	1.23	0.87	1.16
	(0.89, 1.04)	(1.08, 1.4)	(0.79, 0.97)	(1, 1.34)
2012	0.73	0.74	0.73	0.73
	(0.63, 0.84)	(0.58, 0.93)	(0.59, 0.88)	(0.55, 1.04)
2013	1.17	1.16	1.26	1.26
	(1.01, 1.35)	(0.9, 1.49)	(1.04, 1.51)	(0.95, 1.65)
2014	1.16	1.19	1.22	1.34
	(1, 1.35)	(0.91, 1.52)	(1.01, 1.46)	(1.01, 1.77)
2015	1.03	1.00	1.15	1.08
	(0.89, 1.19)	(0.77, 1.3)	(0.96, 1.38)	(0.83, 1.42)
2016	0.82	0.89	0.75	0.99
	(0.69, 0.97)	(0.66, 1.2)	(0.6, 0.92)	(0.71, 1.36)
σ_β_	3.4	26.3	2.3	18.5
Φ	1.11	4.27	0.77	3.91
N	5978	268	5978	268

*Notes:* Data from the National Justice Databse and several public police departments. Values in parentheses give 95% credible intervals

Specifically, we estimate that at the tract level, Black residents are 3 times more likely to experience force than are White residents and about 3.6 times more likely to experience force at the police precinct level. We do not estimate that residents are significantly more likely to experience force at the tract level but do find that they may be slightly (1.23 times) more likely to experience force at the police precinct level. In terms of injury, when we aggregate data at the Census tract level, we estimate that Hispanic residents are slightly less likely (.87 times) to experience force than Whites. However, when we aggregate data to the police precinct, we do not estimate a statistically significant difference between injury rates between Whites and Latinx people.

## Discussion

Our goal was to investigate whether police interactions with PwSMI resulted in greater or fewer uses of force and injuries than would be expected based on a population-level measure of exposure. To accomplish this, we combined a unique data set with public police behavioral data and merged them with census tract level synthetic estimates of the share of the population with serious mental illness. The resulting analyses revealed that PwSMI are 12 times more likely to experience use of force and 10 times more likely to experience injuries from that force than persons without serious mental illnesses. The magnitude of the disparity was several times larger than estimated Black-White disparities in police use of force. Our estimates were robust to changes in the size of the area estimated, with models of both census tract and police precinct revealing similar outcomes.

We believe the study advances both the scientific understanding of how police engage PwSMI and the practical consideration of how police engage the increasing number of mental health crises they are called to resolve. While raw disparities in police contact are not always an indication of individual-level officer bias, they provide a useful metric through which to analyze how individual, situational, and systemic factors affect the rates at which different populations experience police force while controlling for an underlying measure of exposure [22, 23]. In this context, a measure of relative disparities in use of force may be a useful metric as some police departments attempt to reduce number of contacts and incidents of force police have with PwSMI while the population prevalence of serious mental illness simultaneously increases [24, 25]. Thus, while cities and police departments may make significant investments in policies aimed at reducing police use of force against PwSMI raw rates of force or injury incidents may reflect greater prevalence of SMI rather than ineffectual policies. We suggest that police reform measures implemented in some US cities designed to reduce incidents of force—such increased investments in public health and the redirection of emergency 911 calls related to mental health away from police and towards more specialized service providers—should consider such a disparity measure to evaluate the success of these programs.

Despite the robustness of our models to size of area, there are still significant limitations that merit attention for future research.

The first limitation is that we must rely on imperfect instruments: the police and the NCS-R. Because there is no objective metric for serious mental illness available to patrol officers, we used data based on officers’ assessments of suspects during or shortly after an encounter. While there is evidence that officers are no worse than trained mental health professionals at diagnosing individuals at a glance, officer judgments are surely imperfect and may well include systematic errors [11, 12].

The NCS-R is also limited. This survey excludes non-English speaking heads of households from the sample and may also systematically undercount individuals from certain racial or ethnic backgrounds due to socio-cultural differences in reporting mental health symptoms [26]. The NCS-R was also fielded from 2001 to 2003 while both the data on police use of force and ACS data used to generate synthetic estimates of the population with SMI were drawn from 2011 to 2016. If the correlates of SMI have changed in that period then the precision of our estimates of the prevalence of SMI in Census tracts and police precincts and our associated estimates of disparities in force and injury events may be affected.

Second, our inability to estimate certain demographic elements limits the generalizability of these findings. In terms of homelessness, though estimates of serious mental illness among homeless populations vary, some have concluded that rates of SMI among homeless populations are as high as 40% [27, 28]. We include supplementary analysis with an estimate of what city-level overrepresentation looks like when incorporating city-level homeless estimations in an online Additional file 1. While PwSMI are still overrepresented at the city level when the homeless population is accounted for, future research would benefit greatly from an inclusion of more granular data on homeless populations and their rates of SMI.

Finally, our study relies on a nonrandom sample of nine police departments. The sample police departments are not nationally representative and accordingly, our ability to generalize our results nationally are limited.

## Conclusions

The past few years have seen significant public and academic interest in police reforms that would reduce use of force against vulnerable and non-White populations. While evidence of racial disparities in police use of force in America are relatively well established, significantly less is known about disparities across other dimensions of vulnerability, such as mental health. This can be attributable both to limitations in gathering data on the mental health status of justice-involved persons and estimating the population prevalence of SMI. The present study provides evidence that PwSMI are significantly overrepresented in police use of force and suspect injury events. Across cities in our sample, the relative risk that PwSMI experience force and injury is large: several times higher than the risk that Black and Latinx persons relative to White persons. Future work should investigate the relative risk of police contact and force for PwSMI across a larger sample of studies or the effect that police reforms or police interventions can have on reducing disparities in force.

## Supplementary Information

**Additional file 1.**

## Data Availability

The data that support the findings of this study are available from the six anonymous police departments, but restrictions apply to the availability of these data, which were used under license for the current study, and so are not publicly available. Data are however available from the authors upon reasonable request and with permission of partnering police departments. Data from Dallas, New Orleans, and Los Angeles are available from the authors upon request. Interested authors should contact Dr. Ayobami Laniyonu at a.laniyonu@utoronto.ca.

## Abbreviations

PwSMI: People/person with serious mental illness
SMI: Serious mental illness
NCS-R: National Comorbidity Survey, Replication
