# Supplementary material for: Measuring disparities in police use of force and injury among persons with serious mental illness
Source: BMC Psychiatry. 2021 Oct 12;21:500. doi: 10.1186/s12888-021-03510-w (PMC8513301; doi:10.1186/s12888-021-03510-w)
Supplement: Supplementary file 1 — Additional file 1. [file 12888_2021_3510_MOESM1_ESM.docx]

**Supplementary Appendix**

*Descriptive statistics from NCS-R*

**Table S1:** Variable coding and frequency from NCS-R (N = 5,692)

| **Variable** | **Frequency** |
| --- | --- |
| *Age* |  |
| 18-20 (reference) | 347 |
| 21-24 | 451 |
| 25-34 | 1141 |
| 35-44 | 1258 |
| 45-54 | 1123 |
| 55-64 | 663 |
| 65+ | 655 |
| *Gender* |  |
| Male (reference) | 2382 |
| Female | 3310 |
| *Race* |  |
| White (reference) | 4180 |
| Asian | 83 |
| Black | 717 |
| Hispanic | 527 |
| Other | 185 |
| *Marital Status* |  |
| Never Married (reference) | 1217 |
| Divorced, Separated, or widowed | 1239 |
| Married | 3236 |
| *Education* |  |
| Below high school (reference) | 856 |
| High school | 1709 |
| Some college | 1707 |
| Bachelors | 806 |
| Some post-Bachelors | 614 |
| *Employment Status* |  |
| Not in Labor Force | 1630 |
| Employed | 3766 |
| Unemployed | 283 |
| *Poverty Index* |  |
| 0 | 362 |
| 1 | 815 |
| 2 | 861 |
| 3 | 763 |
| 4 | 725 |
| 5 | 2166 |

Estimates of raw disparities accounting for city level homelessness rates

**Figure S1**: City-level disparities in use of force among persons with serious mental illness accounting for city level estimates of the homeless population


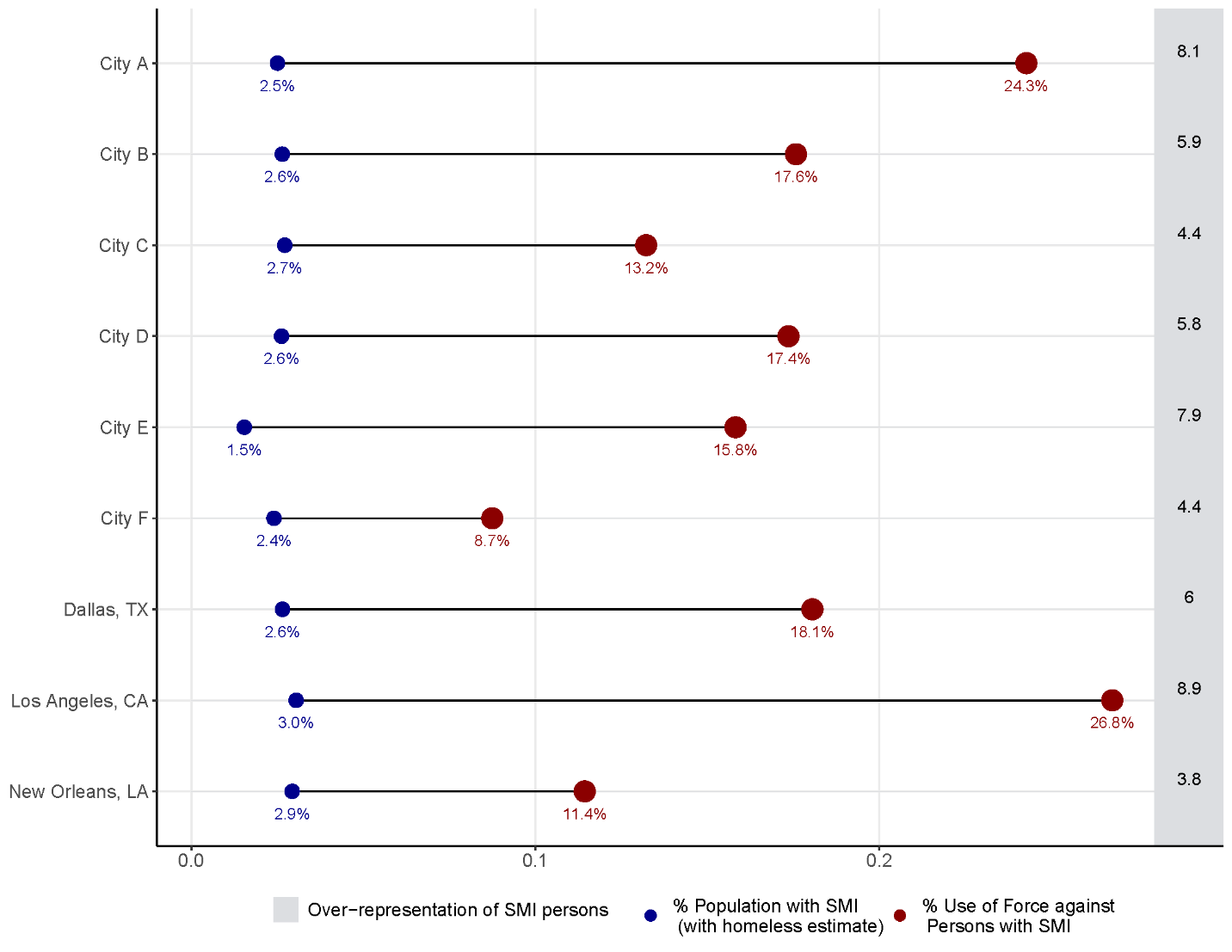


*Note***:** We re-calculate raw city-level disparities in police use of force against PwSMI accounting for estimates of the homeless population in each city. Specifically, for each city in our data set, we pull corresponding point-in-time estimates of the homeless population for that year. Since point-in-time estimates of the local homeless population do not routinely measure the share of the homeless population with SMI, we conservatively estimate a large rate of SMI among the homeless at 42.3% ^1^.

**Figure S2:** City-level disparities in injury among persons with serious mental illness accounting for city level estimates of the homeless population


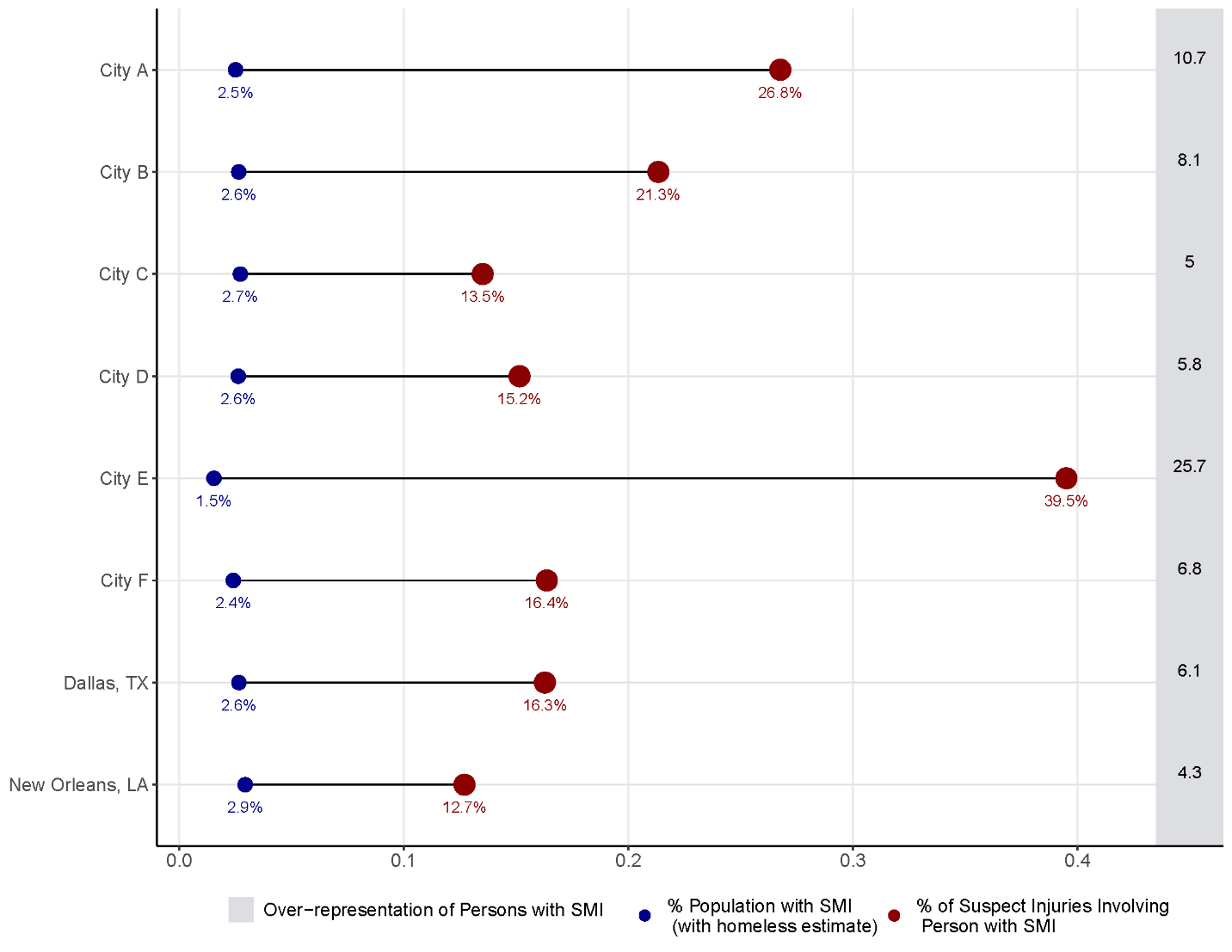


*Note***:** We re-calculate raw city-level disparities in injury among PwSMI accounting for estimates of the homeless population in each city. Specifically, for each city in our data set, we pull corresponding point-in-time estimates of the homeless population for that year. Since point-in-time estimates of the local homeless population do not routinely measure the share of the homeless population with SMI, we conservatively estimate a large rate of SMI among the homeless at 42.3%.^1^

**Figure S3:**  Posterior distribution for estimated coefficients and convergence plots for tract-level model – use of force


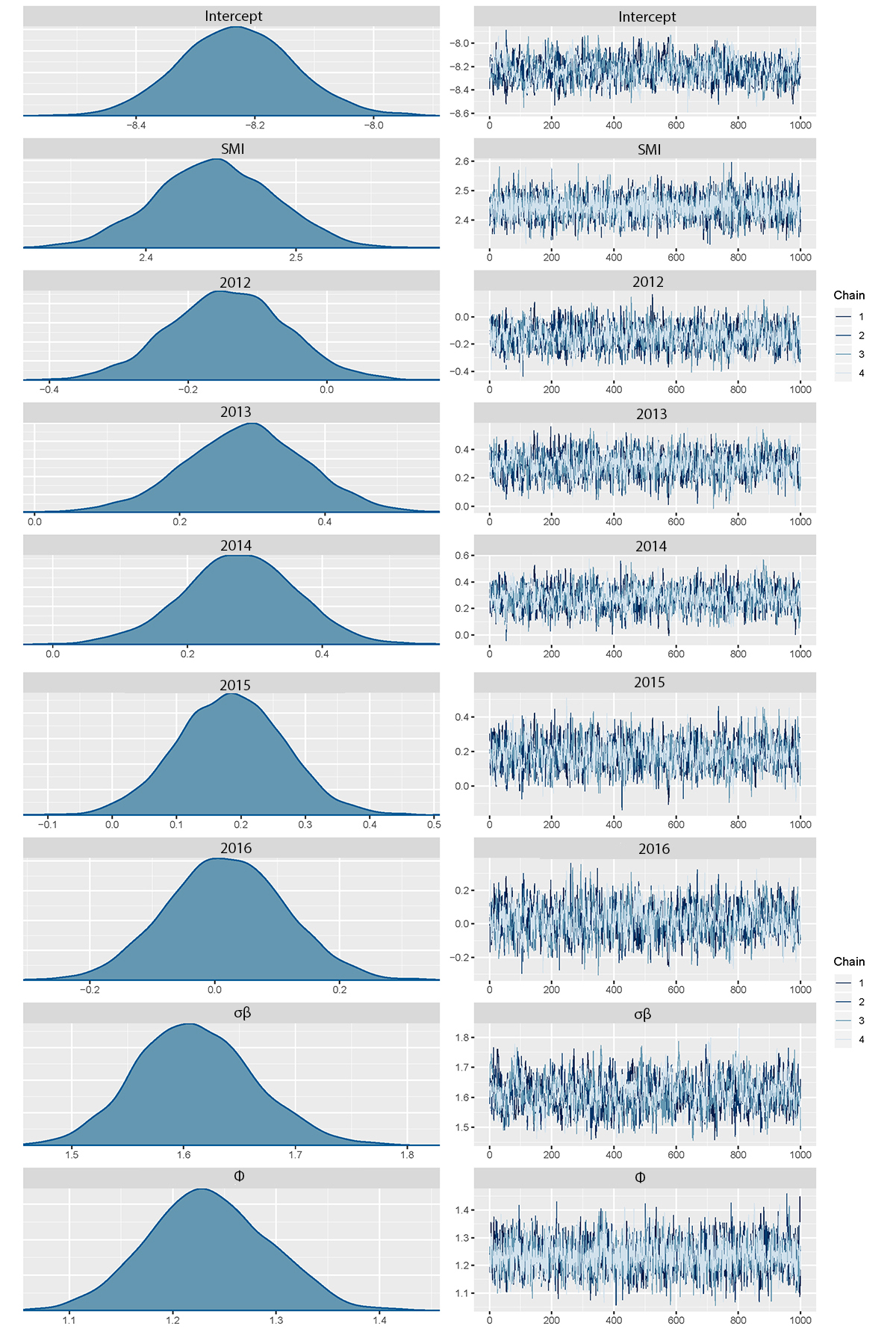


**Figure S4:** Posterior distribution for estimated coefficients and convergence plots for tract-level model – suspect injuries


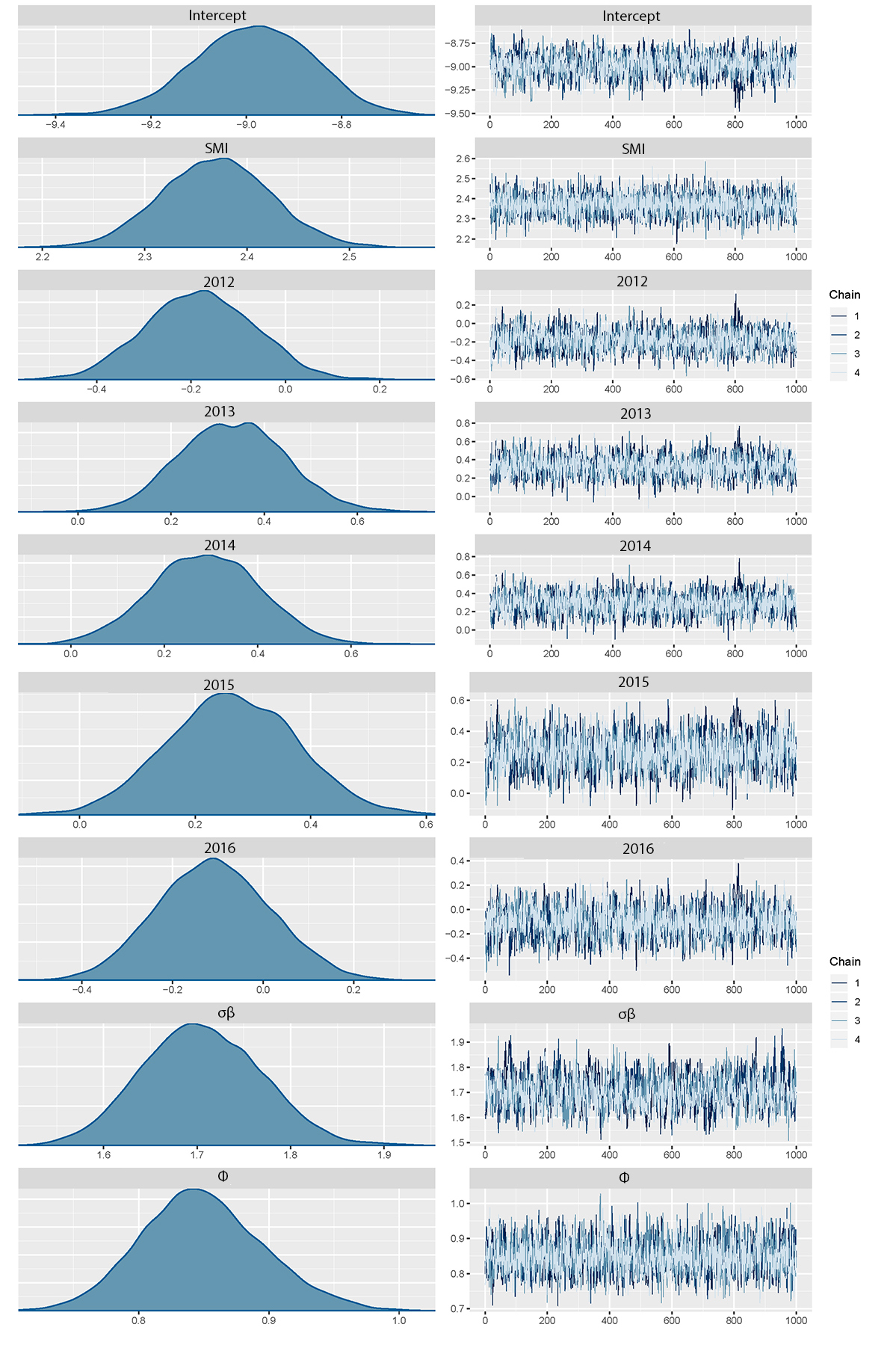


**Figure S5:** Posterior distribution for estimated coefficients and convergence plots for precinct-level model – use of force


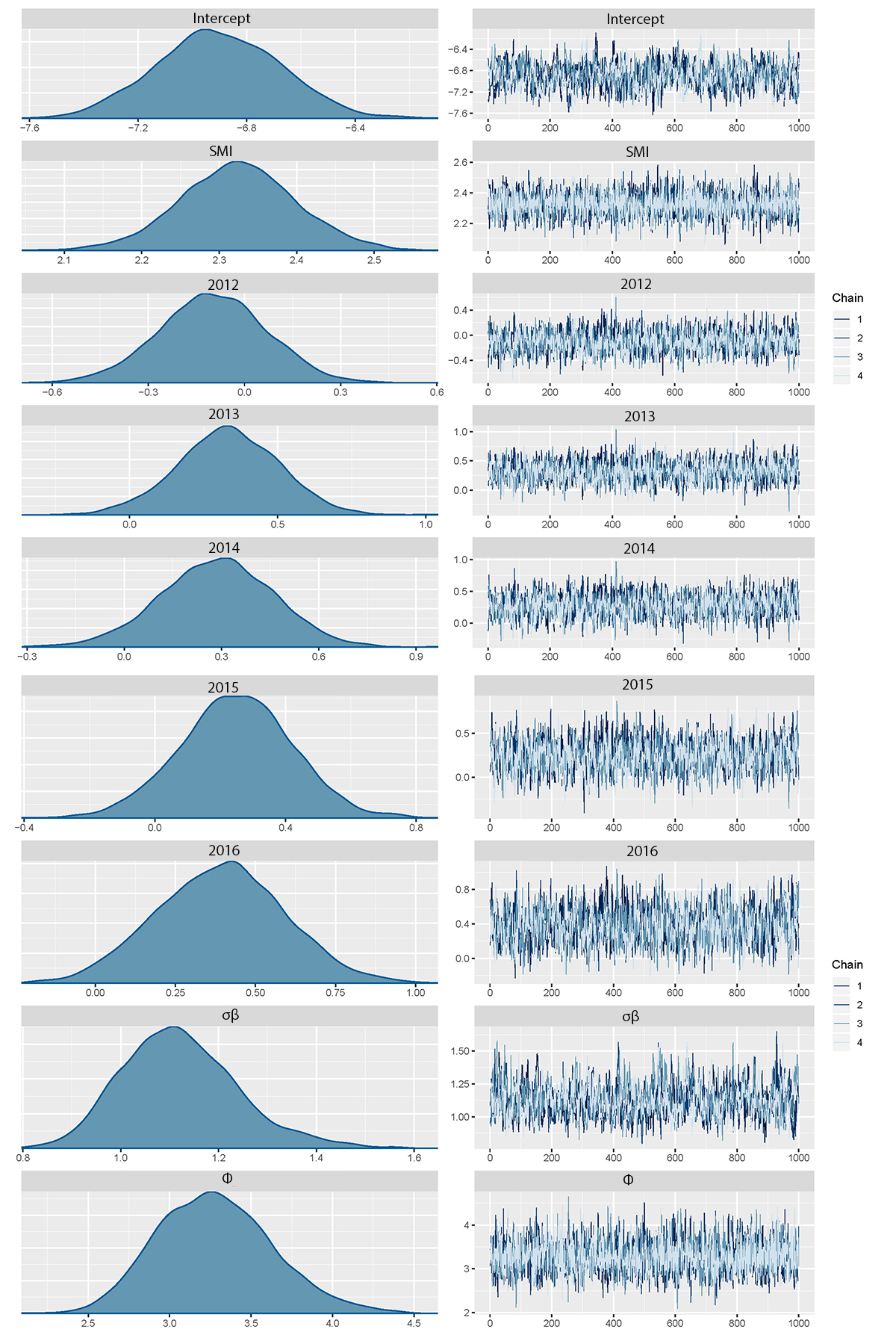


**Figure S6:** Posterior distribution for estimated coefficients and convergence plots for precinct-level model – suspect injuries


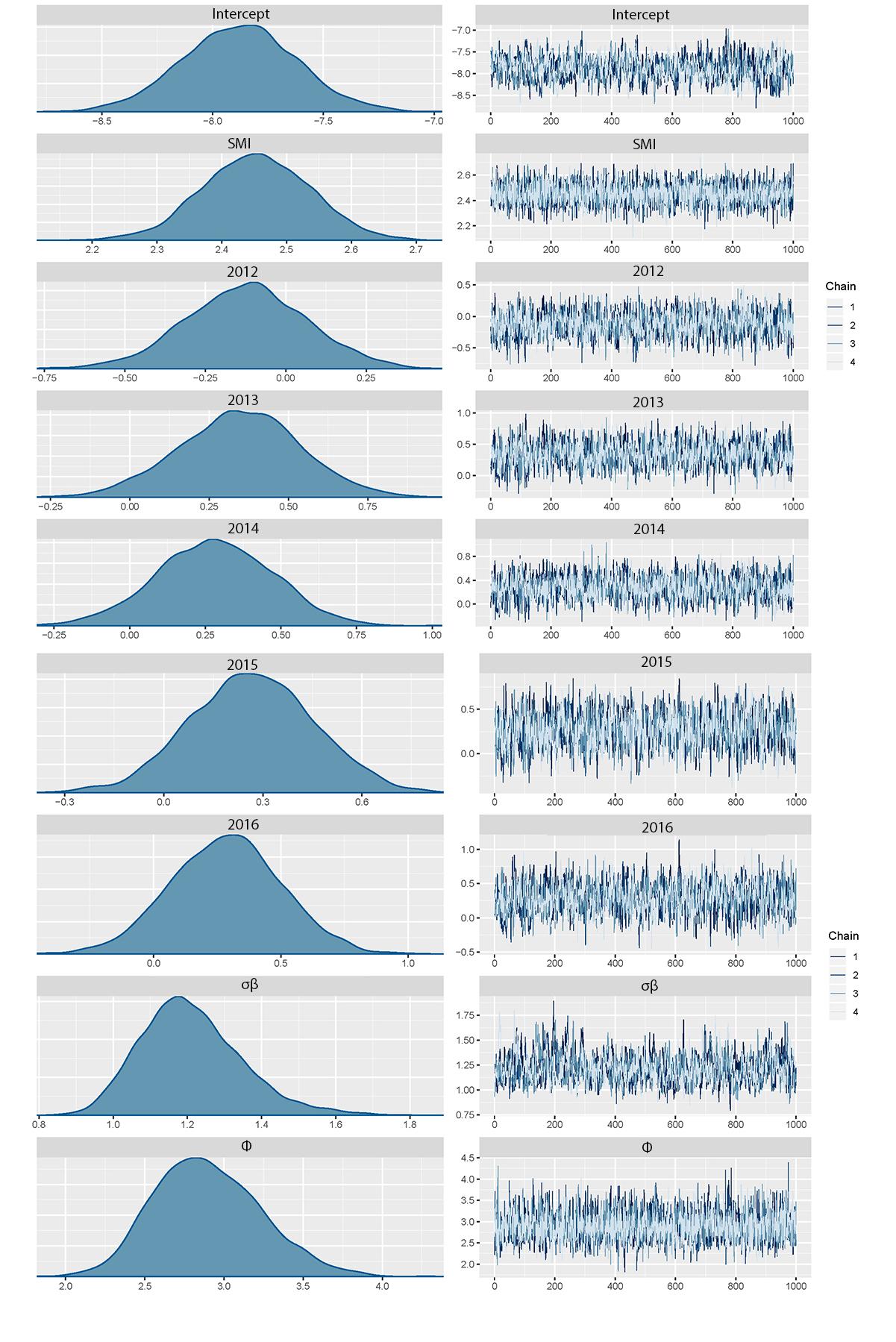


**References**

1. Fazel S, Khosla V, Doll H, Geddes J. The prevalence of mental disorders among the homeless in western countries: systematic review and meta-regression analysis. *PLoS medicine*. 2008;5(12):e225.
